# Predictability and stability of laser-assisted subepithelial keratectomy with mitomycin C for the correction of high myopia

**DOI:** 10.1097/MD.0000000000007076

**Published:** 2017-06-02

**Authors:** Lawrence P.L. Iu, Michelle C.Y. Fan, Ivan N. Chen, Jimmy S.M. Lai

**Affiliations:** a Department of Ophthalmology, The University of Hong Kong, Grantham Hospital; b Department of Ophthalmology, Hong Kong Sanatorium and Hospital, Hong Kong.

**Keywords:** anterior surface ablation, LASEK, laser-assisted subepithelial keratectomy, myopia, refractive, surgery

## Abstract

The purpose of this study was to evaluate the predictability and stability of laser-assisted subepithelial keratectomy (LASEK) with mitomycin C (MMC) in correction of high myopia (≤−6.0 diopters [D]) as compared to low-to-moderate myopia (>−6.0 D).

This is a retrospective, comparative, cohort study which included 43 eyes of 43 consecutive patients who underwent LASEK with MMC in a private hospital in Hong Kong by a single surgeon. Twenty-five eyes had high myopia (mean spherical equivalent [SE] = −8.53 ± 1.82 D) and 18 eyes had low-to-moderate myopia (mean SE = −3.99 ± 1.37 D) before surgery.

In terms of refractive predictability, mean SE was significantly better in eyes with preoperative low-to-moderate myopia than high myopia at 6 months (0.04 ± 0.23 vs 0.31 ± 0.52 D, *P* = .035). In terms of refractive stability, between 1 and 3 months, both groups had mean absolute change of SE of around 0.25 D. Between 3 and 6 months, preoperative low-to-moderate myopia group had significantly less absolute change of SE compared to high myopia group (0.07 vs 0.23 D, *P* = .003). More eyes with preoperative high myopia changed SE by more than 0.25 D than those with low-to-moderate myopia between 3 and 6 months (32.0% vs 5.6%, *P* = .057).

In conclusion, LASEK with MMC is more unpredictable and unstable in correction of high myopia than low-to-moderate myopia. The refractive outcome of most low-to-moderate myopia correction stabilizes at 3 months. Stability is not achieved until after 6 months in high myopia correction.

## 1 Introduction

Laser-assisted in-situ keratomileusis (LASIK) is currently the most commonly performed corneal refractive surgery.^[1]^ In LASIK, a corneal stromal flap is created and lifted before laser ablation is performed to correct refractive error. The need of a corneal stromal flap limits the amount of stromal tissue available for laser ablation, which in turn restricts the amount of refractive error that can be corrected. On the other hand, laser-assisted subepithelial keratectomy (LASEK) is a surface ablation technique which creates only a thin epithelial flap with the aid of diluted alcohol,^[2,3]^ and therefore preserves more stromal tissue for laser ablation. As a result, high degree of refractive error can be corrected and risk of postoperative ectasia is reduced.^[3]^ LASEK is the treatment of choice for patients with high refractive error, thin cornea, and history of recurrent corneal erosion syndrome.^[3]^ Mitomycin C (MMC) is an antiproliferative agent used to reduce the risk of corneal haze formation after surface ablation operation.^[3,4]^

The efficacy and safety of LASEK in correcting myopia have been shown in previous studies.^[5–9]^ The result in high myopia correction is not very reliable because after large amount of tissue ablation, significant wound healing and corneal remodeling occurs leading to unpredictable and unstable refractive changes.^[10,11]^ Enhancement surgery will be necessary if the result is deviated from intended refraction and is dissatisfactory to patients. It is important to wait until the refraction has become stable before enhancement surgery is performed.^[12]^ However, the time required for stability to be achieved after LASEK is not known. Whether stability would be achieved earlier in low myopia correction than high myopia correction has not been investigated before. Therefore, this study was performed to evaluate the predictability and stability of LASEK with MMC to correct high myopia (≤−6.0 diopters [D]) , as compared to low-to-moderate myopia (>−6.0 D), and to determine the time when refractive outcome is stable.

## 2 Methodology

This is a retrospective study in which all consecutive patients who received LASEK with MMC at the Hong Kong Sanatorium and Hospital by one single surgeon (author INC) for the correction of myopia between January 2011 and December 2012 were reviewed. Only subjects who had posttreatment follow-up for at least 6 months were included. Subjects were excluded if they had history of refractive surgery on the cornea, preexisting corneal or retinal diseases that could affect visual outcome, and nonplano target refraction. Only the right eyes of each patient were included for analysis to avoid 2-eye correlation bias. The study was approved by the institutional review board at the Hong Kong Sanatorium and Hospital, and conducted according to the Declaration of Helsinki.

Complete ophthalmological examination was performed in all subjects before the operation, which included best corrected visual acuity (BCVA), uncorrected visual acuity (UCVA), manifest refraction, slit-lamp biomicroscopy, central corneal thickness, tonometry, and dilated fundal examination by indirect ophthalmoscopy. All subjects were followed up at 1 day, 1 week, 1 month, 3 months, and 6 months after operation. Slit-lamp biomicroscopy was performed in all follow-up visits to examine the corneal status. Manifest refraction, BCVA, and UCVA were assessed at 1 month, 3 months, and 6 months after the operation.

Eligible eyes were divided into 2 study groups according to preoperative refraction for analysis: low-to-moderate myopia group with spherical equivalent (SE) >−6.0 D and high myopia group with SE ≤−6.0 D. The primary outcome measures included stability and predictability of postoperative refraction at 6 months. Secondary outcome measures included safety and efficacy.

Predictability was evaluated by mean SE (sphere plus half cylinder power) and mean absolute SE (absolute value of SE) at 6 months. Absolute SE measured the absolute amount of refractive deviation and eliminated the problem in averaging positive and negative SE results. Stability was evaluated by measuring SE refractive change and absolute SE refractive change, between 1 and 3 months, between 3 and 6 months, and the proportion of eyes that had changed by >0.25 D. Efficacy was evaluated by comparing the postoperative UCVA and preoperative BCVA. Efficacy index was calculated by the ratio of mean postoperative UCVA to mean preoperative BCVA in decimals. Safety was evaluated by the drop of BCVA at 6 months from baseline, proportion of eyes that lost 2 or more lines and the frequency of complications. Severity of corneal haze was defined according to O’Keefe et al.^[3]^ Safety index was calculated by the ratio of mean postoperative BCVA to mean preoperative BCVA in decimals.

### 2.1 Surgical procedure

The procedure was performed under topical anesthesia. The cornea was marked with a trephine. A sponge soaked with 20% alcohol was applied to the corneal surface for 20 to 60 seconds for de-epithelialization. The loosened epithelium within the delineated area was separated to create an epithelial flap. The corneal surface was rinsed with balanced salt solution copiously to remove all alcohol and then dried completely with sponge and suction before laser ablation. Surface ablation was performed with excimer laser by WaveLight Allegretto Wave or WaveLight EX500 machine. MMC was applied to the ablated bed with sponge for 30 to 60 seconds to prevent haze formation. The corneal surface was rinsed with balanced salt solution copiously. The epithelial flap was repositioned. Bandage contact lens was applied at the end of operation. Postoperatively patients were prescribed topical antibiotics and steroids. Lubricants were prescribed as required. The patient was followed at 1 day, 1 week, 1 month, 3 months, and 6 months after the operation.

### 2.2 Statistical analysis

SPSS was used to perform the statistical analysis (IBM SPSS V.24). All demographic data were expressed as mean ± standard deviation. BCVA and UCVA were converted to logarithm of the minimum angle of resolution (logMAR) units for statistically analysis. Differences in BCVA and UCVA were measured by 2-sample *t* test. Difference in SE was measured by Mann–Whitney *U* test. The change of SE between 1 and 3 months, and that between 3 and 6 months were evaluated by Wilcoxon signed ranks test. Categorical variables were compared with Chi-square test, and Fisher exact test was used when the expected frequency of more than 10% of cells in a table was less than 5. *P*-value of <0.05 was considered as statistically significant. All tests were 2-sided.

## 3 Results

A total of 46 consecutive patients received LASEK with MMC between January 2011 and December 2012. Three patients were excluded because they did not have follow-up at 3 months, leaving 43 eyes from 43 patients eligible for analysis (53% male, mean age 29.2 ± 6.6 years, range 20–53 years). Eighteen eyes belonged to low-to-moderate myopia group (mean SE = −3.99 ± 1.37 D), and 25 eyes belonged to high myopia group (mean SE = −8.53 ± 1.82 D). There were no significant differences between two groups in terms of baseline BCVA and central corneal thickness. (Table 1) The optical zone of treatment was 6.5 mm for low-to-moderate myopia eyes and 6.0 – 6.5 mm for high myopia eyes.

**Table 1:**
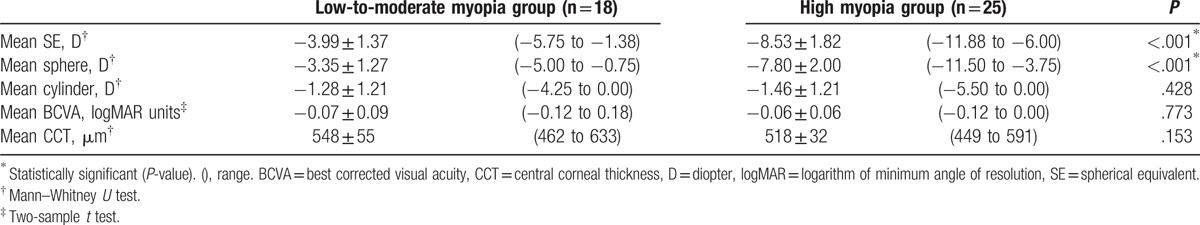
Preoperative data.

### 3.1 Predictability

The mean SE of residual refractive error was -0.03 D, +0.01 D and +0.04 D in low-to-moderate myopia group and +0.23 D, +0.34 D and +0.31 D in high myopia group at 1, 3 and 6 months respectively after operation. (Table 2) The differences between 2 groups were statistically significant at 3 and 6 months (*P* = .013 and *P* = .035, respectively).

**Table 2:**
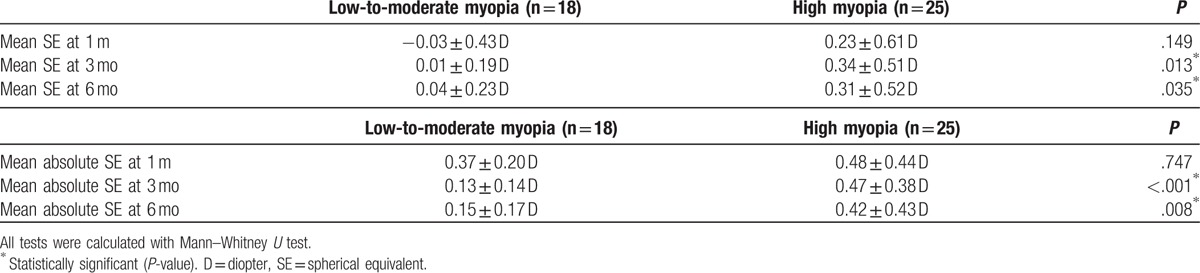
Predictability outcome.

The mean absolute SE was 0.37, 0.13, and 0.15 D in the low-to-moderate myopia and 0.48, 0.47, and 0.42 D in the high myopia group at 1, 3, and 6 months respectively after operation. (Table 2) The differences between 2 groups were statistically significant at 3 and 6 months (*P* < .001 and *P* = .008, respectively).

**Figure 1:**
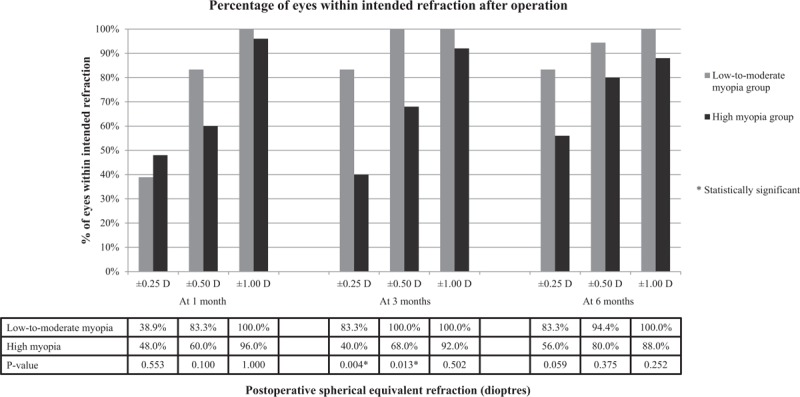
Percentage of eyes achieving specified levels of intended refraction after surgery.

**Figure 2:**
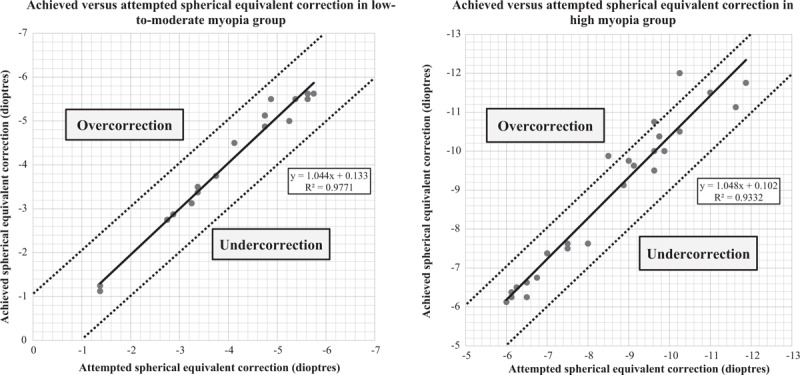
Scattered plot and linear regression analysis between achieved and attempted spherical equivalent (SE) refractive correction in low-to-moderate myopia group (left) and high myopia group (right).

The proportion of eyes which achieved ±0.25 and ±0.50 D of intended refraction was significantly higher in the low-to-moderate myopia group than high myopia group at 3 months (83.3% vs 40.0%, *P* = .004 and 100.0% vs 68.0%, *P* = .013, respectively) (Fig. 1). The proportion of eyes which achieved ±0.25 and ±0.50 D of intended refraction was higher in the low-to-moderate myopia group than high myopia group at 6 months but the differences were not statistically significant (83.3% vs 56.0%, *P* = .059 and 94.4% vs 80.0%, *P* = .375 respectively) (Fig. 1). No eyes in the low-to-moderate myopia group and 3 eyes in the high myopia group failed to achieve ±1.00 D of intended refraction at 6 months (Fig. 2).

### 3.2 Stability

**Figure 3:**
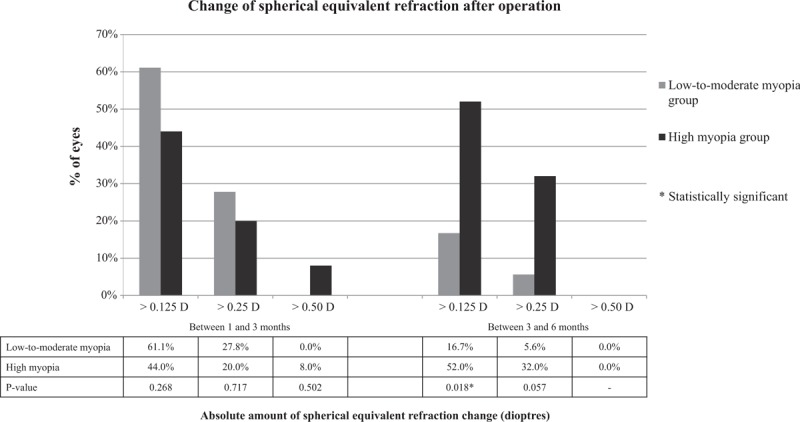
Percentage of eyes with different amounts of absolute spherical equivalent (SE) refractive change between 1 and 3 months and between 3 and 6 months after surgery.

Table 3 indicates that between 1 and 3 months, the mean absolute SE changed by 0.24 ± 0.19 D in low-to-moderate myopia group and 0.22 ± 0.24 D in high myopia group. The difference was not statistically significant between 2 groups (*P* = .376). Between 3 and 6 months, the mean absolute SE changed by 0.07 ± 0.12 D in the low-to-moderate myopia group and 0.23 ± 0.18 D in high myopia group. The difference was statistically significant between 2 groups (*P* = .003). In addition, there were more eyes in high myopia group than in low-to-moderate myopia group which changed SE by more than 0.125 D (52.0% vs 16.7%, *P* = .018) and by more than 0.25 D (32.0% vs 5.6%, *P* = .057) between 3 and 6 months (Fig. 3).

**Table 3:**
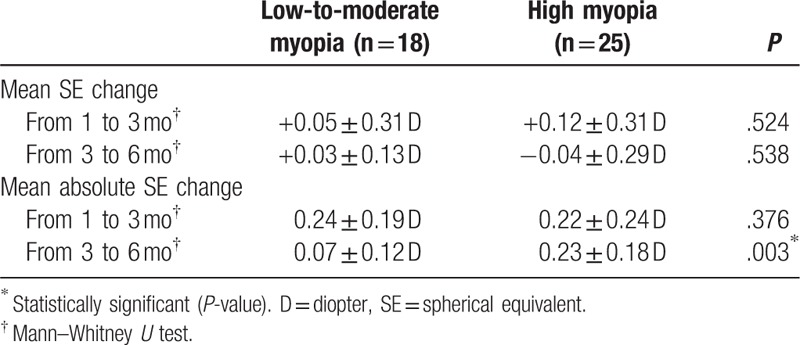
Stability outcome.

### 3.3 Efficacy

The mean UCVA was 0.04, −0.03, and −0.04 logMAR units in low-to-moderate myopia group and 0.07, 0.01, and 0.01 logMAR units in high myopia group at 1, 3, and 6 months, respectively (Table 4). The differences between 2 groups were not statistically significant at all follow-up visits.

**Table 4:**
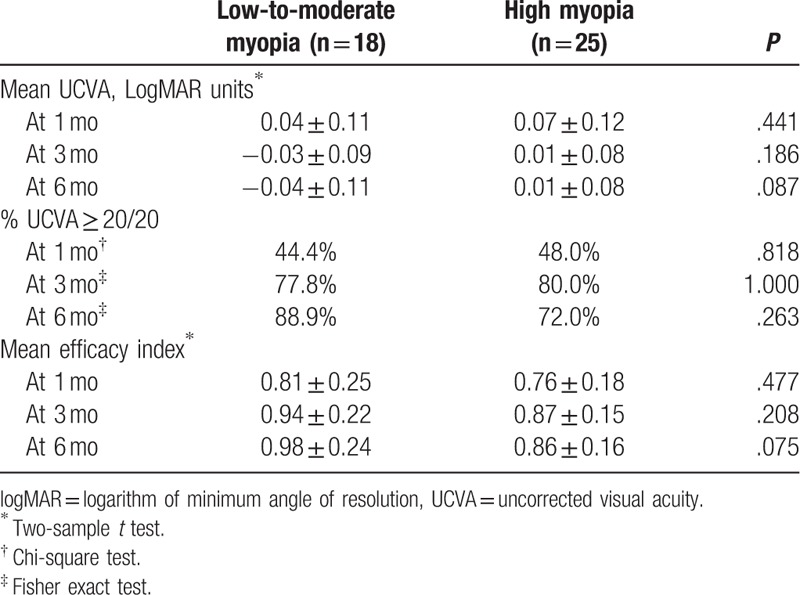
Efficacy outcome.

There were similar proportions of eyes which had UCVA ≥20/20 in low-to-moderate myopia group and high myopia group at 1 months (44.4% vs 48.0%), 3 months (77.8% vs 80.0%), and 6 months (88.9% vs 72.0%) (Table 4). The differences between 2 groups were not statistically significant at all follow-up visits.

The efficacy indexes were 0.81, 0.94, and 0.98 in the low-to-moderate myopia group and 0.76, 0.87, and 0.86 in the high myopia group at 1, 3, and 6 months, respectively (Table 4). The differences between 2 groups were not statistically significant at all follow-up visits.

### 3.4 Safety

The mean BCVA was −0.03, −0.05, and −0.08 logMAR units in low-to-moderate myopia group and 0.02, −0.04, and −0.06 logMAR units in high myopia group at 1, 3, and 6 months, respectively (Table 5). The differences between 2 groups were not statistically significant at all follow-up visits.

**Table 5:**
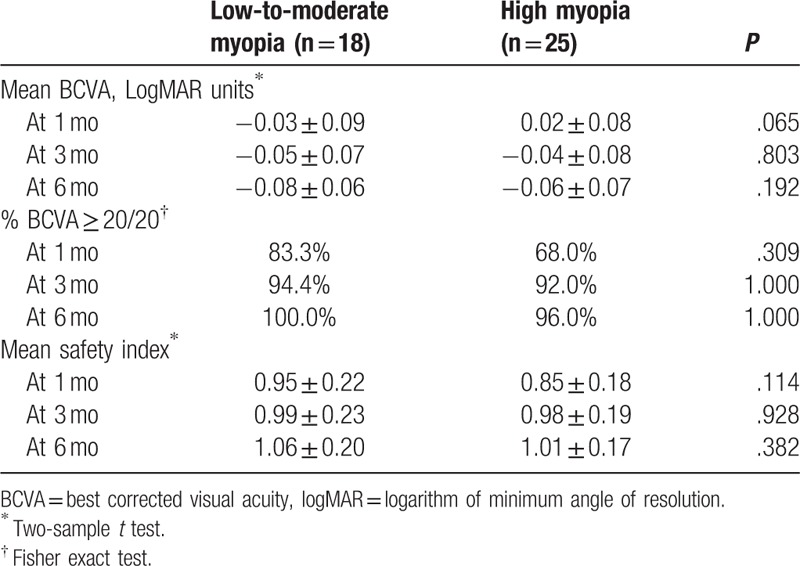
Safety outcome.

All eyes (100%) in low-to-moderate myopia group and 96.0% of eyes in high myopia group had BCVA ≥20/20 at 6 months. The safety indexes were 0.95, 0.99, and 1.06 in the low-to-moderate myopia group and 0.85, 0.98, and 1.01 in the high myopia group at 1, 3, and 6 months, respectively (Table 5). The differences between 2 groups were not statistically significant at all follow-up visits. An increase of safety index with time was noted in both groups.

All surgeries were uneventful without intraoperative complications. There were no problems with corneal epithelial healing. Only 1 eye in the high myopia group with preoperative SE −9.625 D developed corneal haze after surgery; the corneal haze was of grade 1 and BCVA was 20/15 at 6 months. No eyes experienced drop of 2 lines or more of BCVA at 6 months.

## 4 Discussion

Our study showed that LASEK with MMC was more unpredictable and unstable in the correction of high myopia ≤−6.0 D than low-to-moderate myopia >−6.0 D. We observed that high myopia correction had a hyperopic shift of more than 0.25 D at 3 and 6 months after surgery, and only 80.0% were within ±0.50 D of intended refraction at 6 months. On the contrary, low-to-moderate myopia correction had a hyperopic shift of less than 0.125 D at 3 and 6 months after surgery, and 94.4% were within ±0.50 D of intended refraction at 6 months. Our results were comparable to previous studies, which showed that in high myopia correction 79% were within ±0.50 D of intended refraction at 6 months,^[13]^ while in low-to-moderate myopia correction 85% to 92% were within ±0.50 D of intended refraction at 6 months after LASEK.^[7,14]^

Early studies suggested LASEK was stable for low to high myopia correction by comparing the overall postoperative SE at different time points after operation.^[5,7,8]^ For example, in one study the authors reported the overall SE changed from −0.09 D at 1 month to −0.25 D at 4 months and −0.15 D at 6 months after LASEK.^[7]^ This method to predict stability could be biased because positive SE change in 1 patient and negative SE change in another patient would offset the overall measured changing effect. Therefore, in this study we evaluated stability by measuring the absolute change of SE to eliminate the problem of balancing positive and negative changes. We showed that the absolute change of SE between 1 and 3 months was similar in the low-to-moderate myopia group (0.24 ± 0.19 D) and high myopia group (0.22 ± 0.24 D), and that between 3 and 6 months was significantly more in the high myopia group (0.22 ± 0.24 D) than in low-to-moderate myopia group (0.07 ± 0.12 D).

The major mechanism resulting in refractive instability and unpredictability after refractive surgery was due to corneal healing.^[10]^ After laser ablation, keratocyte-mediated regrowth of ablated stroma occurs leading to refractive changes and myopic regression.^[10]^ In higher myopia correction, deeper stromal ablation was necessary which was followed by more regeneration of corneal stroma and resulted in more instability and unpredictability.^[10]^

Enhancement surgery might be necessary if the refractive result is dissatisfactory. It is usually recommended to postpone enhancement surgery until refraction has been stable to achieve optimal result and avoid over-treatment.^[12]^ In general, a refraction which changed by less than 0.25 D measured at least 1 month apart is considered stable.^[12,15]^ In our study, both groups had around 0.25 D of absolute SE change between 1 and 3 months, suggesting refractive stability was not achieved before 3 months. Between 3 and 6 months, high myopia correction continued to have around 0.25 D of absolute SE change, and 32.0% changed SE by more than 0.25 D. On the contrary, low-to-moderate myopia correction had only 0.07 D of absolute SE change between 3 and 6 months, and only 5.6% changed SE by more than 0.25 D. This suggests majority of low-to-moderate myopia correction stabilizes at 3 months, and stability could not be achieved until after 6 months in high myopia correction.

The disadvantages of LASEK, as compared to LASIK, include more postoperative pain, slower visual recovery, and higher risk of corneal haze formation in high myopia correction.^[2,16]^ Corneal haze developed as a result of keratocytes proliferation, migration, and differentiation into myofibroblasts after ablation.^[4]^ The risk of corneal haze increased with higher degree of refractive correction.^[17]^ In our study, corneal haze was only a rare event and the severity was mild and did not affect final visual acuity. A final BCVA of at least 20/20 was achieved in all eyes with low-to-moderate myopia and 96% of eyes with high myopia.

Photorefractive keratectomy (PRK) and epipolis LASIK (epi-LASIK) are alternative surface ablation techniques for patients with thin cornea and high refractive error. In PRK, the epithelium is removed mechanically prior to laser ablation without repositioning.^[3]^ Because the epithelium was not preserved in PRK, there will be direct contact between the ablated stroma and the inflammatory mediators in tear film leading to significant keratocytes activation and haze formation.^[18]^ Corneal haze had been shown to be more common in PRK than LASEK in the early postoperative period in previous studies.^[19]^

In epi-LASIK, a specially made microkeratome was used to create an epithelial flap which is repositioned after laser ablation.^[2]^ In contrast to producing a cleavage plane within the basement membrane in LASEK, epi-LASIK produces a cleavage plane beneath the basement membrane and therefore preserves basal epithelial structures from laser ablation.^[20]^ Epi-LASIK was thought to produce less inflammation and has better visual recovery compared to LASEK because it preserves the basal membrane structures and avoids the use of alcohol which was potentially toxic to epithelial cells.^[21]^ However, most studies failed to show any differences between LASEK and epi-LASIK in safety, efficacy, epithelial healing time, and corneal haze formation.^[14,22,23]^ On the other hand, one previous study suggested LASEK provided faster visual rehabilitation and had better safety and efficacy than epi-LASIK at 3 months after operation.^[24]^

One approach which might improve the stability of refractive result is to perform concurrent corneal collagen cross-linking with refractive operations. Previous study showed that LASIK combined with prophylactic corneal collagen cross-linking had better predictability and stability than LASIK alone.^[25]^ Evidence from large randomized controlled studies is still lacking. Another study showed that LASEK combined with prophylactic corneal collagen cross-linking had less refractive change than LASEK alone but the difference was not statistically significant.^[26]^

Small-incision lenticule extraction (SMILE) is a corneal refractive surgery which, instead of creating a stromal flap as in LASIK, uses femtosecond laser to create an intrastromal lenticule.^[27]^ The lenticule is then removed through a small peripheral wound to reduce corneal thickness and refractive power to treat myopia.^[27]^ Compared with LASIK, SMILE has the advantages of avoiding corneal flap complications, less high order aberrations, less corneal denervation, and less dry eye syndrome.^[27]^ The efficacy and safety paralleled that of LASIK.^[27]^ One retrospective study of 45 eyes with high myopia showed that 94% were within ±0.5 D of target at 12 months after SMILE.^[28]^ The major limitations of SMILE include lack of eye-tracker system to correct cyclotorsion and unsatisfactory results for hyperopic corrections.^[27,29]^

High myopia is generally more common in the Chinese population than in Caucasian population. In the Chinese population, a prospective study of 37,932 eyes showed that LASIK had refractive predictability within ±0.5 D of target in around 75% of eyes with myopia between −5.0 and −10.0 D and around 65% of eyes with myopia greater than −10.0 D at 3 months after operation.^[30]^ In another study of 274 high myopic eyes of Chinese, 58% to 60% were within ±0.5 D of target at 3 months after LASIK.^[31]^ The refraction changed by less than 1.0 D in 78% to 84% eyes at 3 months.^[31]^ In a prospective comparative study of Chinese, predictability within ±0.5 D of target was achieved in 97% among 34 eyes after SMILE and 94% among 32 eyes after PRK.^[32]^ Phakic intraocular lens (pIOL) insertion is an option for extreme myopia correction. One retrospective study of 63 eyes of Chinese showed that 97% were within ±0.5 D of target at 6 months after pIOL.^[33]^ Although pIOL was shown to have a good safety and efficacy indices, there was risk of endothelial cell loss.^[33]^

There were several limitations in this study. First, the follow-up period was limited to 6 months only, and long-term stability and predictability after 6 months could not be assessed. Second, due to the retrospective design this study was limited to assessment of visual acuity and refractive outcome only. We could not evaluate patients’ satisfaction, visual function, degree of postoperative pain, and any visual disturbances after surgery. Similarly, we could not investigate the changes of cornea morphology, including corneal topography and corneal hysteria, which might explain the instability of refractive result. Third, the sample size was small, and there were no comparative groups of LASIK, SMILE, or PRK to compare the safety and efficacy between different refractive surgeries. Finally, MMC was applied over a range of 30 to 60 seconds, and we could not rule out possibility of different degrees of instability arising from different durations of MMC exposure. Despite these limitations, this study had the strength of direct comparison between high myopia and low-to-moderate myopia correction. Absolute value of SE change and proportion of patients with different levels of SE change were measured to evaluate stability more accurately. Since this study consisted of 1 single surgeon only, it had the strength of reduced surgical techniques variability.

In conclusion, LASEK with MMC is more unpredictable and unstable in correction of high myopia than low-to-moderate myopia. The refractive outcome of most low-to-moderate myopia correction stabilizes at 3 months. Stability is not achieved until after 6 months in high myopia correction.
